# Exploring the ‘January effect’ at a university hospital in Pakistan: a retrospective cohort study investigating the impact of trainee turnover on patient care quality outcomes

**DOI:** 10.1186/s12909-023-04708-0

**Published:** 2023-10-16

**Authors:** Qamar Riaz, Rida Mitha, Muhammad Shahzad Shamim, Qurat-Ul-Ain Virani, Asim Belgaumi, Muhammad Rizwan Khan, Rozina Roshan, Nida Zahid, Adil Haider

**Affiliations:** 1 Department for Educational Development, Aga Khan University Hospital, Karachi, Pakistan; 2 Department of Surgery, Section of Neurosurgery, Aga Khan University Hospital, Karachi, Pakistan; 3 Aga Khan University Hospital, Karachi, Pakistan; 4 Department of Surgery, Aga Khan University Hospital, Karachi, Pakistan; 5 Department of Infection Prevention & Hospital Epidemiology (DIPHE), Aga Khan University Hospital (AKUH), Karachi, Pakistan

**Keywords:** Trainee turnover, Patient care quality outcomes, July effect

## Abstract

**Objective:**

There are reports of a potential rise in a teaching hospital’s morbidity and mortality rates during the trainee turnover period, i.e., with the induction of new residents and house staffs, and the changeover of clinical teams. The published literature displays mixed reports on this topic with lack of reproducible observations. The current study was conducted to explore existence of any such phenomenon (January effect) in Pakistan.

**Methods:**

This retrospective cohort study was conducted at Aga Khan University Hospital, Karachi, Pakistan. Five-year (2013–2018) record of all the patients in all age groups related to these outcomes was retrieved and recorded in specifically designed questionnaire. Different outcome measures were used as indicators of patient care and change in these outcomes at the time of new induction was related to possible January effect.

**Results:**

During the five-year study period, more than 1100 new trainees were inducted into the post graduate medical education program (average of 237 per year) with more than 22,000 inpatient admissions (average of 45,469 per year). Some patterns were observed in frequencies of surgical site infections, medication errors, sentinel events, patient complaints, and adverse drug reactions. However, these were not consistently reproducible and could not be directly attributed to the trainee turnover. All other indicators did not show any pattern and were considered inconclusive. No effect of overlap was observed.

**Conclusions:**

Inconsistency in the patient care quality indicators do not favor existence of January effect in our study. Further research is recommended to establish our results.

## Background

It is commonly believed that the quality of health care declines during trainee turnover at the start of the academic year, compared to the rest of the training continuum [1]. The rise in a teaching hospital’s reported morbidity and mortality rates during the turnover period has been associated with the induction of new, less experienced residents and house staff, and the changeover of clinical teams. This phenomenon has been referred to as ‘July effect’ in US [1], and ‘August killing season’ in UK [2] based on the months when these transitions predominantly occur in these countries. In medical literature, the terms ‘July effect’ and ‘August killing season’ highlight the potential challenges concerning patient care quality and safety associated with the turnover of trainees at the beginning of the academic year.

There are mixed reports in peer-reviewed literature on this topic both in terms of whether such an effect does exist, as well as the indicators that may be studied to verify the existence of such an effect. The various indicators studied so far include, medication errors, mortality, morbidity, near misses, adverse events, and efficiency [3, 4]. A systematic review on the topic concluded “Mortality increases and efficiency decreases in hospitals because of year-end changeovers, although heterogeneity in the existing literature does not permit firm conclusions about the degree of risk posed, how changeover affects morbidity and rates of medical errors, or whether particular models are more or less problematic” [5]. Another study done in a tertiary care pediatric hospital reported that while there was an increase in reported medical errors involving pediatric residents in July compared with the months surrounding July, there was no difference in numbers of adverse events from those errors between these months [6]. In short, it is unclear if there is an effect on the patient outcomes as a result of the health care staff turnover and which circumstances, procedures, patient types, or institutions might be more influenced.

The training framework in Pakistan differs from most other countries due to the presence of an extra mandatory ‘House Job’ or ‘Internship’ year, which every doctor must complete immediately after graduating from medical school. This year long training requires rotations through both medical and surgical specialties, despite the graduates’ specific clinical area of interest.

Aga Khan University Hospital (AKUH) is Pakistan’s only JCIA accredited academic medical centre. AKUH has one of the country’s largest and most sought-after post-graduate training program, that includes a year-long internship, residency, and fellowship programs. In addition, AKUH also has a sought-after school of nursing and nursing education services. The academic year for internship, residency, fellowship programmes, as well as for trainee nurses begins from January. Prior to 2015, the turnover of new inductees has been like that in most programmes where the old batch leaves and the new cohort of inexperienced fresh graduates steps in. Although the existence of any January effect at AKU has never been formally studied, in 2015 an overlap of 3 days for interns was introduced, i.e., for three full days, the passing out interns and recently enrolled interns worked on the floor together. This was done because of an exercise involving focused interviews and small group discussions with graduated and chief residents who suspected increased medical errors and disruptions in service provision [7].

This study was conducted to confirm if any such phenomenon exists at AKU, and whether the introduction of overlap made any difference. To the best of our knowledge, no such study has been conducted in this region prior to this one.

## Methods

This retrospective cohort study was conducted on an open cohort at Aga Khan University Hospital, Karachi, Pakistan. All records related to patients admitted in wards irrespective of age group, were retrieved from the office of the Chief Medical Officer (CMO) for five years i.e., from 2013 to 2018, and recorded using specifically designed questionnaires. Outcome measures that were used as indicators of patient care for this study included: hospital incidence of adverse drug reactions, medication errors and near miss, sentinel events, surgical site infections (SSI), hospital incidence of multiple drug resistant organisms (MDRO), quality of medical record documentation, needle stick and sharps injuries to health care staff (NSI), health care staff exposure to contaminants, wrong labeling of site/ specimen, patient satisfaction reports, and patient complaints. This data is routinely collected and compiled as per hospital’s ongoing quality improvement process as defined in Table 1. Clinical areas where interns or first year residents do not rotate, or are not credentialed for patient care, such as intensive care units, coronary care units, recovery rooms, operating rooms, etc., were excluded from the study. Since, this study is a retrospective cohort, this may lead to an information bias.

**Table 1:** Operational Definitions of the Patient Care Outcome Measures

Patient-care indicator	Operational definition
Adverse drug reaction	An injury caused by taking medication where a causative relationship can be shown
Medication error	Any error in the prescribing, dispensing, or administration of a drug, irrespective of whether such errors lead to adverse consequences or not, are the single most preventable cause of patient harm
Adverse event	An injury caused by medical management rather than by the underlying disease or condition of the patient
Near miss	An unplanned event which did not result in injury, illness, or damage – but had the potential to do so
Sentinel events	Any unanticipated event in a healthcare setting resulting in death or serious physical or psychological injury to a patient or patients, not related to the natural course of the patient’s illness. (American healthcare accreditation organization)
Surgical site infection (SSI)	An infection that occurs after surgery in the part of the body where the surgery took place

Analysis: Descriptive analysis for each indicator including mean, standard deviation (SD), frequencies and percentages were calculated using SPSS statistical software (SPSS) version 20.0. The data was compiled for four quarters of each year.

Quarter 1: January-March.

Quarter 2: April-June.

Quarter 3: July-September.

Quarter 4: October-December.

A pattern showing decline in the first quarter of the year which is also the time when new postgraduate trainees are inducted in the university, could be attributed to the turnover of trainees or the ‘January effect’.

Ethical considerations: For all the data, only the total number was retrieved; individual patient records were not retrieved or reviewed to maintain the anonymity and confidentiality of any patient, health care staff or trainees. An exemption was obtained from the institutional ethics review committee (AKU-ERC) before beginning the study.

## Results

During the five-year study period, an average of 237 new interns, residents and fellows were inducted into the post graduate medical education program each year with an average of 45,469 inpatient admissions per year, making this one of the largest samples to have been studied for this phenomenon. Significant results of this study included noticeable pattern changes in frequencies of surgical site infections, medication errors, sentinel events, patient complaints and adverse drug reactions. All other indicators did not show any pattern and were considered inconclusive.

**Fig. 1:**
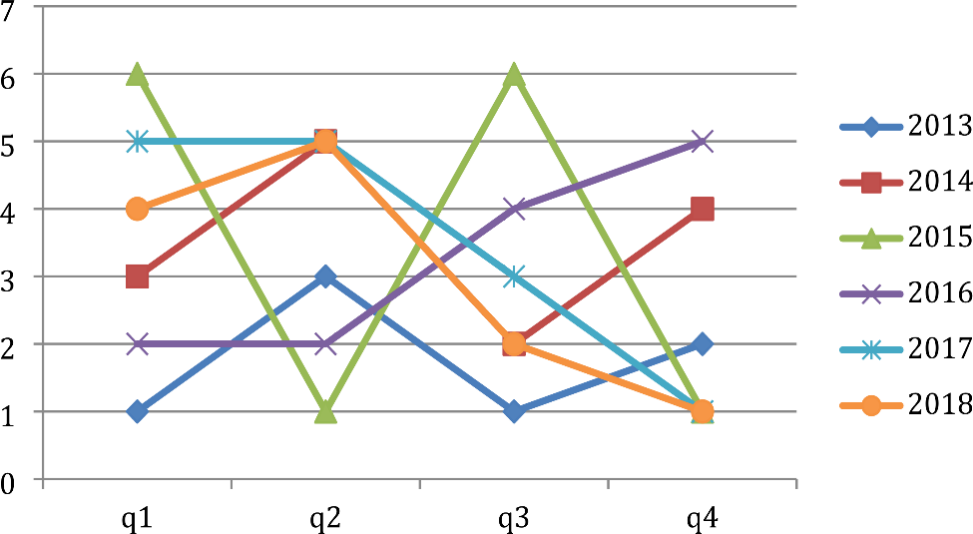
Frequency of Sentinel Events

**Fig. 2:**
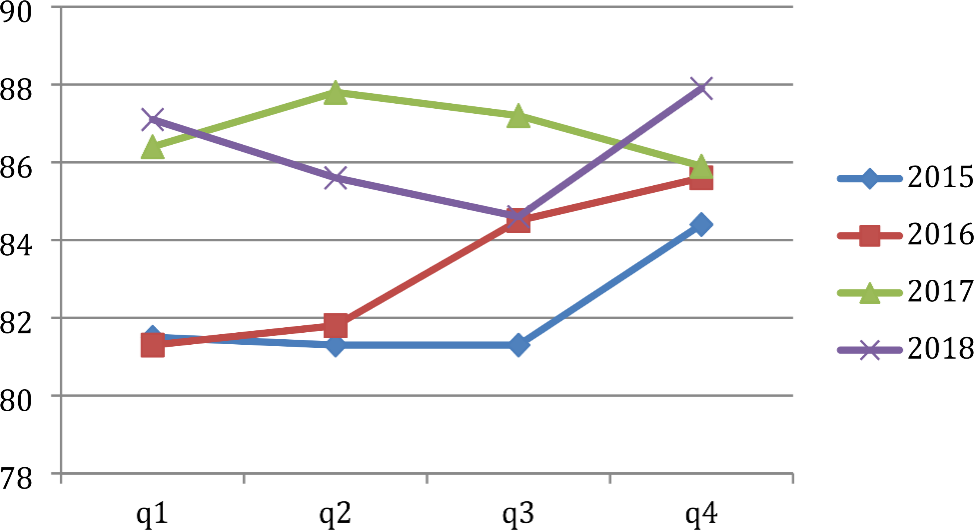
Frequency of Patient Complaints

Apart from the year 2017, frequency of sentinel events at our hospital remained relatively higher in the first quarter of the year as compared to the previous year’s fourth quarter (Fig. 1). Similarly, the frequency of patient complaints also increased in the first quarter of a new year as compared to the previous year’s fourth quarter (Fig. 2). Given the very small number of sentinel event (1–6 per quarter) and patient complaints (81–88 per quarter) compared to the large volume of patients (more than 45,000 per year), these differences are not statistically significant. However, these two were the only variables that consistently showed a January effect.

The rate of surgical site infections was shown to rise in the first quarter of all the five years 2014–2018 as compared to the fourth quarter of the previous year. Interestingly, for three of these years (2014, 2017, and 2018) the peak increase in surgical site infection rates were actually in the second quarter. Frequency of adverse drug reactions was only seen to increase in the first quarter of the years 2016 and 2018, and these years also had a peak increase in the frequency of adverse drug reactions in the second quarter. Percentage of medication errors showed an increase in the first quarter of the years 2014 and 2015. There was, however, a decreasing percentage of medication errors throughout the years 2013–2015 and an increasing percentage of medication errors seen in the years 2016–2018. The frequencies of SSI, medication error, and adverse drug reaction are shown in Table 2.

**Table 2:** Frequency of Patient Care Outcome Indicators

	Q1	Q2	Q3	Q4
	**Frequency of Medication Error (%)**			
**2013**	0.07	0.1	0.04	0.04
**2014**	**0.09**	0.14	0.13	0.05
**2015**	**0.09**	0.08	0.09	0.07
**2016**	0.04	0.07	0.02	0.06
**2017**	0.04	0.03	0.02	0.05
**2018**	**0.02**	0.05	0.04	0.03
	**Frequency of Surgical Site Infections (%)**			
**2013**	2.1	1.5	0.2	0.6
**2014**	**3.8**	3.8	3.5	3.2
**2015**	**3.4**	2	0.9	0.6
**2016**	**1.6**	1.7	1.8	1.2
**2017**	**2.2**	3.6	1	0.9
**2018**	**2.9**	3.4	2.6	1.8
	**Frequency of adverse drug reactions (n)**			
**2013**	31	38	51	38
**2014**	29	40	45	25
**2015**	18	41	34	40
**2016**	**50**	78	53	37
**2017**	29	30	18	22
**2018**	**36**	73	46	47

## Discussion

This is the first study that explored the association between trainee turn over and patient care quality outcomes or what may be called as ‘January effect’ at a University Hospital in Karachi, Pakistan. Beginning of every academic year in a teaching hospital is faced with multiple challenges, the most important being the acclimatization of the fresh medical graduates or junior trainees and residents to the newer role in providing patient care [8]. This phenomenon commonly has a threatening connotation, often known as the ‘July effect’ in different parts of the world, mainly because of the general perception of increased medical errors by the junior or newly inducted trainees which results in comprised patient care [1]. The literature clearly traces the deterioration in patient quality and safety and increase in the incidence of preventable medical errors to new interns and residents as they advance towards mastering patient care [5]. Several varying parameters have been used to study this phenomenon such as length of hospital stay, duration of procedures, hospital charges, fatal medical errors, mortality rate, postoperative complications, efficiency in the Emergency department [5, 9].

Our analysis showed a marginal increase in the sentinel events and patient complaints reported in the first quarter compared to the rest of the year. The difference in actual numbers was however too small to draw meaningful conclusions. We also found an increase in the frequency of surgical site infections and adverse reactions in the first quarter of every year as compared to the previous year. This could be an indirect indicator of the possible ‘January effect’ in our set up as the fresh inductees take up responsibilities in the first quarter of every year while the experienced ones graduate in the last quarter of the previous year. This is in agreement with a large number of higher-quality studies that have consistently found greater evidence of a true “July effect” throughout training in many disciplines including surgery, orthopedics, pediatrics, neurosurgery, obstetrics and gynecology, and oral surgery for various procedures across the world [10, 11]. In addition to the induction of inexperienced newbies, studies have also identified increase in the responsibilities of the trainees promoted to the next level of training as the underlying cause for the ‘July effect’ [12].

Our study showed a spike in the surgical site infections and adverse reactions in the second quarter for three years that we studied. This could be because of the lowering down of the compensatory defense mechanism of the hospital staff i.e., senior physicians and nurses who may be otherwise more vigilant in supervising the new inductees and following the protocols in the earlier months of induction. Studies have shown that more senior members of the medical team, including fellows and attending surgeons, as well as the nursing staff provide a greater level of support to the fresh trainees to ensure continuity of care and quality standards [13]. Also new interns and residents are more cautious in the beginning of the academic year in following the standardized management protocols and seek help more often when required instead of trying to solve problems themselves [1]. The possible decline in the deliberate measures once the trainees have settled in the new system, may have resulted in the peak in the second quarter. However, the possibility of seasonal effect on the frequency of SSI cannot be over-ruled. Anthony et al., identified warm weather as the most important factor responsible for surgical site infections [14] and the second quarter showing the peak in SSI, marks extreme warm weather in Pakistan.

While most of the studies reported the existence of a July effect, there are also studies that negated the induction of new trainees as the major cause for poor patient outcomes or offered inconclusive findings [15]. Nochure et al. evaluated postsurgical outcomes at a single academic medical center during a 10-year period and concluded that there was no evidence of the July Effect [16]. A study evaluating complications in plastic surgery procedures disproved possibility of July effect [17]. Another study assessing patient outcomes after cardiac surgery denied any effect of new induction, and instead stressed the fundamental role of hospital support systems in safe transition of trainees while ensuring patient outcomes [18]. In our study, the patient outcomes indicators other than the ones mentioned earlier, neither show any specific pattern nor indicate any association with the beginning of the academic year or influx of relatively inexperienced trainees in the hospital.

Literature suggests different measures that could minimize the July effect. One of these is developing an active orientation program, that would improve communication between caregivers, and enhance collegiality between physicians and nurses thus ultimately improving patient outcomes [19]. Literature also emphasizes appropriate senior staff supervision and hospital protocols that would not only prevent the potential for increased complications despite multiple changes occurring within house staff in academic hospitals but also ensure safety and quality of clinical care and learning in teaching hospitals [20].

### Limitations

There are important limitations to our study. Aside from the inherent drawbacks like information bias in using a retrospective data set, we were unable to control for particular factors that may have influenced outcomes and compromise the reliability of the inferences drawn. Because of the way the data was available within the hospital, we were not able to analyze the outcomes for every month that may have diluted or concentrated the results. Also, it did not allow in depth statistical analyses. Finally, this was only a single center study and thus cannot be representative of all academic hospitals in the city and nationally. This calls for high quality researches, possibly prospective cohort studies, to get valid results. Furthermore, likely causes of the deteriorating indicators of patient outcomes needs to be explored further.

### Implications

Despite the gaps in research methodology, our findings have several implications for improving hospital systems and policies related to postgraduate medical education. This stresses the need for (1) a robust orientation for the new inductees focused on education related to hospital polices, systems and medication safety; (2) a more vigorous overlap week where the junior residents can shadow the graduating senior residents thus ensuring enhanced understanding and smooth transition of responsibilities; (3) the hospital also needs to re-evaluate the responsibilities assigned to new interns and residents and the provision of increased supervision; and (4) finally a more robust system of data management system and a system for continuous monitoring.

Incorporating these changes might improve the quality standards for patient care at the hospital by reducing both fatal and non-fatal medication errors, thereby reducing the substantial associated costs as well as enhance the quality of the postgraduate training at the institute.

## Conclusion

This study contributes valuable insights into the “January effect” phenomenon and its potential impact on patient care quality outcomes. The suggested changes could potentially improve patient care quality by reducing medication errors, both fatal and non-fatal, leading to cost savings and enhanced postgraduate training quality at the institution. While certain indicators suggest a possible association, further research and the implementation of proactive measures are necessary to mitigate any adverse effects and ensure the highest standards of patient care in teaching hospitals.

## Data Availability

The datasets used and/or analysed during the current study is available from the corresponding author on reasonable request.
